# The Potential Protective Role of GS-441524, a Metabolite of the Prodrug Remdesivir, in Vaccine Breakthrough SARS-CoV-2 Infections

**DOI:** 10.1007/s44231-022-00021-4

**Published:** 2022-11-09

**Authors:** JiaYi Zhu, Yuchong Li, Jady Liang, Samira Mubareka, Arthur S. Slutsky, Haibo Zhang

**Affiliations:** 1 Keenan Research Centre for Biomedical Science, St. Michael’s Hospital, Unity Health Toronto, Toronto, ON Canada; 2 Department of Physiology, University of Toronto, Toronto, ON Canada; 3 Department of Laboratory Medicine and Pathobiology, University of Toronto, Toronto, ON Canada; 4 The State Key Laboratory of Respiratory Disease, Guangzhou Institute of Respiratory Disease, The First Affiliated Hospital of Guangzhou Medical University, Guangzhou, China; 5 Department of Medical Microbiology and Infectious Disease, Sunnybrook Health Science Centre, Toronto, ON Canada; 6 Interdepartmental Division of Critical Care Medicine, University of Toronto, Toronto, ON Canada; 7 Department of Anaesthesiology and Pain Medicine, University of Toronto, Toronto, ON Canada

**Keywords:** Delta variant, Omicron variant, Non-structural proteins, RNA-dependent RNA polymerase, Variants of concerns

## Abstract

Cases of vaccine breakthrough, especially in variants of concern (VOCs) infections, are emerging in coronavirus disease (COVID-19). Due to mutations of structural proteins (SPs) (e.g., Spike proteins), increased transmissibility and risk of escaping from vaccine-induced immunity have been reported amongst the severe acute respiratory syndrome coronavirus 2 (SARS-CoV-2). Remdesivir was the first to be granted emergency use authorization but showed little impact on survival in patients with severe COVID-19. Remdesivir is a prodrug of the nucleoside analogue GS-441524 which is converted into the active nucleotide triphosphate to disrupt viral genome of the conserved non-structural proteins (NSPs) and thus block viral replication. GS-441524 exerts a number of pharmacological advantages over Remdesivir: (1) it needs fewer conversions for bioactivation to nucleotide triphosphate; (2) it requires only nucleoside kinase, while Remdesivir requires several hepato-renal enzymes, for bioactivation; (3) it is a smaller molecule and has a potency for aerosol and oral administration; (4) it is less toxic allowing higher pulmonary concentrations; (5) it is easier to be synthesized. The current article will focus on the discussion of interactions between GS-441524 and NSPs of VOCs to suggest potential application of GS-441524 in breakthrough SARS-CoV-2 infections.

**Supplementary Information:**

The online version contains supplementary material available at 10.1007/s44231-022-00021-4.

## Introduction

**Fig. 1:**
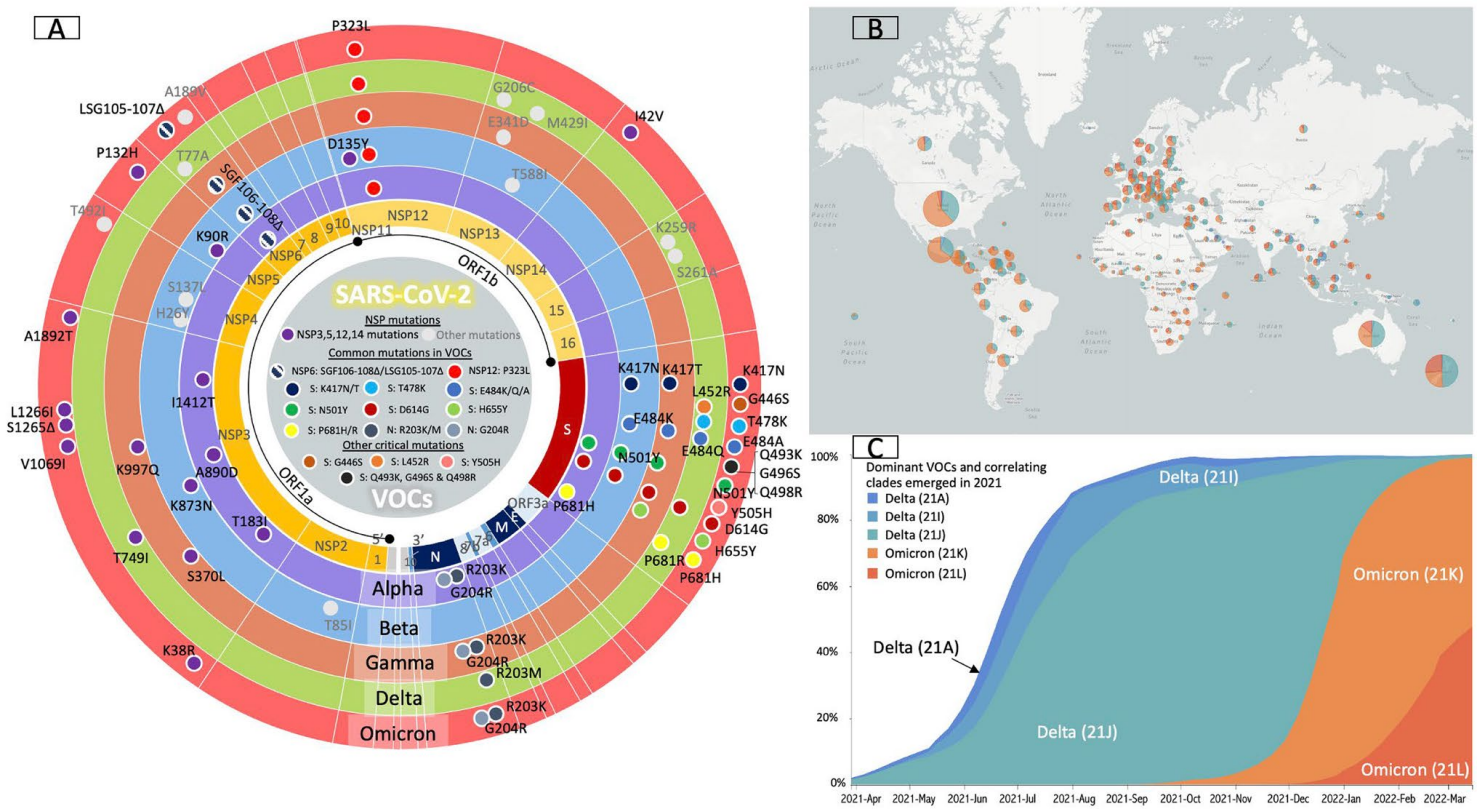
Genome of SARS-CoV-2 and signature mutations of the emerging variants [5, 6, 24]. **A** Genomes of wildtype SARS-CoV-2 and VOCs (Alpha variant (i.e., B.1.1.7); Beta variant (i.e., B.1.351); Gamma variant (i.e., P.1); Delta variant (i.e., B.1.617); and Omicron variant (B.1.1.529)) with indication of the most characteristic mutations, including mutations in ORF1ab, Spike, and Nucleocapsid protein. **B** Global distribution of COVID-19 cases with the currently predominating Delta and Omicron variants. Nextstrain clade label: 21 K indicates Omicron BA.1 lineage; 21L indicates Omicron BA.2 lineage. **C** Frequencies of major Delta and Omicron sub-lineages from April 2021 to March 2022. **B**, **C** Generated using Nextstrain [4, 5]

The severe acute respiratory syndrome coronavirus 2 (SARS-CoV-2) has caused hundreds of millions of cases, and millions of deaths worldwide [1–3]. Recently there has been a surge in cases of COVID-19, largely due to SARS-CoV-2 Variants of Concern (VOCs), including the Alpha (B.1.1.7), Beta (B.1.351), Gamma (P.1), Delta (B.1.617.2), and Omicron (B.1.1.529) variants (Fig. 1 and Supplementary Table S1) [4–11]. The VOCs are potentially more contagious, causing increasingly severe infections, evading the host immune system, and/or inducing reinfections [12]. Notably, the Delta variant has shown a 108% increased risk for hospitalization, 235% increased risk for ICU admission, and 133% increased risk for death compared to the original virus [13]. The Omicron variant has more than double the mutations of the Delta variant and spreads twice as quickly as the Delta variant with higher infectivity and transmissibility [14–17]. Recent data also suggest that the emerging Omicron sub-lineages BA.1 and BA.2 are alarming, due to their increased prevalence worldwide and a higher risk of breakthrough infections than all other VOC lineages caused by substantially reduced vaccine protection and weakened neutralizing antibody responses [16–18]. Growing evidence suggests that reinfections and breakthrough infections may promote the spread of VOCs, especially Delta and Omicron variants [16, 19–23].

There are three different groups of proteins that are encoded in the SARS-CoV-2 genome, namely structural proteins (SPs), non-structural proteins (NSPs), and accessory proteins (APs) [24]. Spike, Nucleocapsid, Envelope, and Membrane proteins are the major SPs (Fig. 1) [24]. With respect to SPs, mutations mainly take place on the receptor-binding domain (RBD) in Spike proteins resulting in changes of its binding affinity to the membrane receptor angiotensin-converting enzyme 2 (ACE2) on host cells [25, 26]. NSPs and APs are more conserved than SPs, yet whose mutational changes may facilitate new pathways involving viral replication and release [24].

Currently available vaccines, including mRNA vaccines (e.g., Pfizer-BioNTech and Moderna) and viral vector-based vaccines (e.g., Janssen and AstraZeneca) can attenuate SARS-CoV-2 and VOC entry into host cells by inducing antibodies targeting RBD of spike protein [27]. However, the VOCs can breakthrough by escaping the target site from vaccine, and may subsequently lead to further mutations [28, 29]. Thus, repurposing and developing antiviral drugs to target the most conserved NSPs of VOCs is an immediate priority [12].

**Fig. 2:**
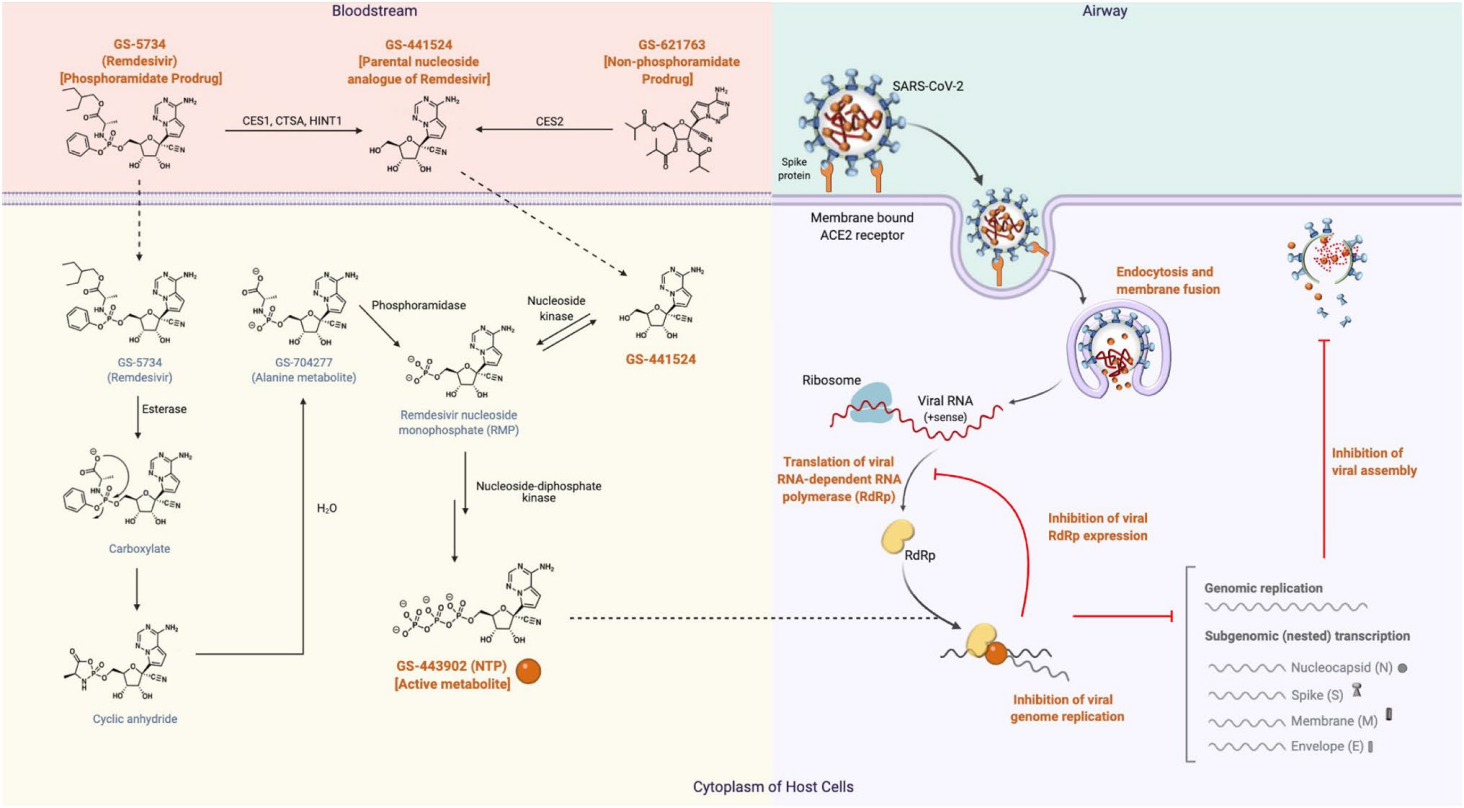
Mechanism of action of GS-441524 and Remdesivir following SARS-CoV-2 infection. The diagram depicts the structures and potential disposition of GS-441524 in SARS-CoV-2 infected cytoplasm of a Type II pneumocyte. Remdesivir is converted into GS-441524 extracellularly and into NTP intracellularly, while the only major metabolic pathway of GS-441524 is its conversion into NTP in cells. The bioactivation of Remdesivir involves liver-specific enzymes Carboxylesterase 1 (CES1), Cathepsin A (CTSA), and Histidine Triad Nucleotide Binding Protein 1 (HINT1). NTP disrupts RdRp activities, inhibits viral replication, and prevents the translation and assembly of viral proteins (e.g., Spike protein, Envelope protein, Membrane protein) to ultimately minimize further damages of the human body from the amplifying SARS-CoV-2 virus. Recreated from “Remdesivir: Potential Repurposed Drug Candidate for COVID-19”, by BioRender.com (2021). Retrieved from https://app.biorender.com/biorender-templates

Antiviral drugs can target different stages of viral replication cycles such as viral entry and fusion, uncoating, transcription, translation, and virion release [30]. In COVID-19, antiviral therapies such as Remdesivir, neutralizing antiviral antibodies (i.e., Casirivimab/Imdevimab), and plasma therapy were granted emergency use by FDA [30–32]. Remdesivir is a nucleoside analogue that was clinically investigated for Ebola virus prior to COVID-19 [30]. As a phosphoramidate, Remdesivir undergoes metabolic transitions to become the active nucleoside triphosphate (NTP; GS-443902) (Fig. 2) [33, 34]. In its metabolically active stage, NTP directly interrupts viral replication by inhibiting viral RNA-dependent RNA Polymerase (RdRp) [33, 35]. Although Remdesivir has exerted some efficacy in treating COVID-19 patients [36], it has shown no decrease in all-cause mortality in patients with severe COVID-19, with most trials demonstrating marginal antiviral benefit [36–39]. On the other hand, GS-441524 is the parent C’-adenosine analogue of Remdesivir, and theoretically has several pharmacological advantages over Remdesivir in the treatment of SARS-CoV-2 infection [28, 40]. In the present article, we focus our discussion on the potential efficacy of GS-441524 as a therapeutic agent in breakthrough SARS-CoV-2 VOC infections [19–21, 23, 41, 42].

## The Non-phosphoramidate GS-441524 Could be Superior Over Remdesivir (Contains the Functional Group Phosphoramidate) Against SARS-CoV-2 and VOCs

Although the catalytic NTP that interacts and interferes with RdRp can be derived from both GS-441524 and Remdesivir, the complicated bioactivation pathway, preferential expression of Remdesivir prodrug bioactivating enzymes in the liver, and short half-life of Remdesivir (~ 1 h) render GS-441524 (~ 3–5 h) a better therapeutic candidate [33, 34, 43]. GS-441524 is bioactivated by nucleoside kinases, which are expressed more evenly across all tissues in the body [33]. Considering that it is quite common to observe comorbidities in patients with severe SARS-CoV-2 infections, GS-441524 could be potentially more potent than Remdesivir and could be an antiviral therapeutic option against SARS-CoV-2 variants with greater patient tolerability.

### Nucleoside Kinase is the Only Enzyme Required for GS-441524 Conversion to Active NTP

The molecular basis of bioactivation demonstrates differences in enzymatic requirement between GS-441524 and Remdesivir. GS-441524 requires only nucleoside kinase for bioactivation [33]. In contrast, Remdesivir requires carboxylesterase 1 (CES1), cathepsin A (CTSA), and histidine triad nucleotide-binding protein 1 (HINT1) that are expressed in kidney and liver tissues to be metabolised involving esterase and phosphoramidase pathways for bioactivation [33, 44, 45]. In severe COVID-19 patients with underlying comorbidities, the liver and kidney are likely to malfunction for the conversion of Remdesivir to bioactive NTP against SARS-CoV-2 (Fig. 2).

### Orally Bioavailable Prodrug of GS-441524

In in vitro studies, GS-621763, an orally bioavailable prodrug of GS-441524, has been shown to have low cytotoxicity and a similar EC_50_ to GS-441524 [46, 47]. Recent studies suggested that administration of GS-621763 is efficacious against SARS-CoV-2 in ferrets and mice [45, 48]. A pharmacokinetic study revealed higher and more consistent plasma concentrations of GS-441524 in ferrets receiving oral GS-621763 compared to those receiving intravenous administration of Remdesivir or GS-441524 [48]. GS-621763 supports the exploration of GS-441524 oral prodrug in the management of breakthrough COVID-19.

### Safety of GS-441524 Over Remdesivir

Data in both cell culture and in animal models indicate that GS-441524 is much less cytotoxic in cells and better tolerated in animals compared to Remdesivir [33, 43, 49, 50]. The latter has shown to induce adverse effects in rhesus macaques (e.g., renal tubular atrophy) and patients (e.g., liver and kidney inflammation) [33, 44]. The non-phosphoramidate GS-441524 may minimise liver and kidney adverse events, enables the drug to be administered in higher doses. The first human study of orally administered GS-441524 for COVID-19 (Trial ID: NCT04859244) in a healthy woman has also shown sustained plasma concentrations and excellent safety profile [51, 52].

### Socio-economic Benefits of GS-4414524 Over Remdesivir

The structural complexity of Remdesivir makes drug production costly and difficult. The minimum production cost of Remdesivir is USD$9.30 for a 10-day treatment course (100 mg two times on Day 1 and 100 mg one time on Days 2–9), which is much more expensive than many other repurposed antiviral drugs for COVID-19 such as fluvoxamine [53–55]. In contrast, GS-441524, with its simpler structure (3 functional groups less than Remdesivir) and as the prodrug of Remdesivir during the production procedure and in tissue metabolism, would be significantly less expensive than Remdesivir to produce [33].

## Combination Therapy of GS-441524

As SARS-CoV-2 variants continue to emerge, there have been increasing interests in developing combination therapies (both virus- and host-targeted) through repurposed drugs, with the goal of better inhibiting viral infections by targeting different mechanistic pathways [56, 57]. As each drug has different yet specific mechanism of action, one advantage of utilizing combination therapies is the potential of achieving drug synergy, offering a treatment that performs better than when administering the individual drugs alone. This also raises the possibility of using lower effective concentrations of each drug in a combination therapy to minimize drug toxicity, side effects, and costs.

### Combination Therapy with Functional Inhibitors of Acid Sphingomyelinase (FIASMA)

FIASMA (e.g., fluoxetine, amiodarone, and imipramine) is a group of psychotropic medications that inhibits the lysosomal enzyme acid sphingomyelinase and regulates the homeostasis of the endolysosomal host–pathogen interface [58, 59]. In in vitro models, FIASMA has been found to efficiently inhibit SARS-CoV-2 entry and propagation via mechanisms such as impairing endolysosomal acidification and inducing cholesterol accumulation within the endosomes [59, 60]. The antiviral potency of FIASMA is further supported by recent clinical studies [55, 58, 61, 62]. For example, in patients with psychiatric disorders hospitalized for severe COVID-19, those receiving FIASMA medications at baseline had significantly reduced risk of intubation or death as compared to those receiving non-FIASMA antidepressants (*p* < 0.01) [58]. Together, these data suggest the potential antiviral potency of FIASMA in treating SARS-CoV-2 infections.

Recently, many papers revealed the synergistic antiviral potential of therapies that combine remdesivir and/or GS-441524 with FIASMA for treating SARS-CoV-2 variants [56, 57, 59]. For example, in in vitro models of SARS-CoV-2, combined therapy of GS-441524 with fluoxetine showed a more superior viral titer reduction (over 99% reduction) than using fluoxetine (60–70% reduction) or GS-441524 treatment alone (90% reduction) [57]. More importantly, the synergistic antiviral effects are not only observed in the SARS-CoV-2 parental strain, but also in the Alpha and Beta variants, providing support for the applicability of such combination treatments for the currently prevailing Omicron variant infections [57]. Omicron variants have also been found to rely heavily on the endocytic pathways for viral entry, which further supports the use of host endolysosome-directed FIASMA with viral replication-directed remdesivir/GS-441524 for treating SARS-CoV-2 infections [63].

### Combination Therapy with Other Drugs

Additionally, combining remdesivir with many non-FIASMA drugs, including itraconazole, baricitinib, and MEK1/2 Inhibitor ATR-002 (Zapnometinib) have also shown synergistic antiviral effects against SARS-CoV-2 [56, 64, 65]. In polarized Calu‐3 cell culture model, itraconazole-remdesivir combination inhibits the production of infectious SARS‐CoV‐2 particles by over 90% and shows synergistic effects [56]. In a double-blind, randomized, placebo-controlled trial, baricitinib-remdesivir treatment reduces recovery time of patients with COVID-19 and accelerates their improvement in clinical status as compared to using remdesivir alone [64]. Treatment combinations of ATR-002 with remdesivir have also been found to display synergistic antiviral effects [65]. The above drugs are promising targets to be used in conjunction with the direct-acting antiviral remdesivir against SARS-CoV-2 and in vivo and clinical studies are critical in further validating their potency.

### Limitations of Combination Therapy

Patients with severe SARS-CoV-2 infections often hold other comorbidities that may be exacerbated when giving additional repurposed drugs or combination therapies [66]. While both GS-441524 and FIASMA have been reported with little adverse effects on organs, careful evaluations need to be taken about the suitability and safety of GS-441524-FIASMA treatments before making a treatment decision [57, 66, 67].

## Interactions Between GS-441524 and VOCs

### Inhibition of RdRp (NSP12, 7, 8) Polymerization

RdRp is a multi-unit transcription complex consisting of NSP12, NSP7, and NSP8, which is essential for the replication of the SARS-CoV-2 genome (Table 1) [35, 68]. NTP is a high-affinity substrate for RdRp through interaction with NSP12 [35, 69] and inhibits viral replication through incorporation by RdRp into nascent viral RNA, predominately resulting in chain termination at the *i* + 3 position with a steric clash of the NTP 1′-CN group with residue R858 of RdRp [70]. The efficient incorporation of NTP into the newly synthesized viral RNA chain is due to the superior selectivity of NTP compared to ATP and other nucleoside analogues [71, 72]. The effective inhibition of RdRp is dependent on the complementarity between NTP and RdRp. When mutations in amino acid residues that interact with NTP are present, the binding properties may be altered to reconstruct the interactions between NTP and RdRp [73].

**Table 1:** Biological functions and GS-441524 interacting residues of NSP12-7–8, NSP5, NSP3, and NSP14

Non-structural protein	Biological functions	GS-441524 interacting residues in NSPs
RdRp (NSP12-7–8) [24, 40, 69, 71, 82]	(1) NSP7 and NSP8 are cofactors of NSP12 (RNA-dependent RNA Polymerase, RdRp), which together form the replication and transcription complex (RTC)	F480, K545, Y546, A547, S549, K551, R553, R555, T556, V557, A558, D618, K621, C622, D623, S682, D761, K798, E811, R836, R858
	(2) NSP12-NSP7-NSP8 complex shows RNA polymerization activity	
	(3) NSP7 mediates single-stranded RNA binding	
	(4) NSP8 is a primase	
NSP3 (i.e., PLpro, papain-like protease) [77, 79, 87]	(1) Key component for coronavirus replication	Hydrogen bonding: I23, N40, F156
	(2) Processes polyprotein (NSP1, 2, 3) via the PLpro domain	Salt-bridges: D22, D157
	(3) Contains a transmembrane domain that is associated with modified host’s endoplasmic reticulum (ER) membranes and is essential for RTC formation	π–π interaction: F156
	(4) Inhibits ubiquitination	vdW interaction: H45, G46*, G47, G48, V49†, L126, S128, G130, I131‡, F132, L153, A154§, V155
NSP5 (i.e., 3CLpro, 3-chymotrypsin like protease; or Mpro, main protease) [81]	(1) Mediates processing of NSPs at 11 distinct cleavage sites, including its own autoproteolysis	Hydrogen bonding: P39, C145, H163, M165
		π–π interaction: H164
	(2) Essential for viral replication and RTC formation	vdW interaction: T25, T26, L27, N28, G29, L30, Y37, C38, P39, H41, M49, C117, Y118, N119, G120, F140, L141, N142, G143, S144, C145, G146, S147, M162, H163, H164, M165, E166
NSP14 [40, 68, 83]	(1) Contains exoribonuclease for proofreading activities	D90, E92, H95‖, N104, F190, E191, D273
	(2) Contains guanine-N7-methyltransferase (N7-MTase) domain to facilitate 5′-cap formation	

^*^, †, ‡, §, ‖: Amino acid residues that have mutations in VOCs reported on Nextstrain as of March 20th, 2022. Each symbol is indicative of one specific mutation. Amino acid residue marked with † had multiple patient samples/isolates reflecting the same mutation. Amino acid residue marked with § had multiple mutations. All the mutations can be referred to source information in Supplementary Table S2

To evaluate the mutations in amino acid residues of major GS-441524-interacting SARS-CoV-2 NSPs (RdRp, NSP3, NSP5, and NSP14), the online database Nextstrain (https://nextstrain.org/ncov/global) was used (up to March 20th, 2022) [4, 5]. As of March 20th, 2022, no mutation in amino acids of RdRp that interact with GS-441524 had been found in VOCs (Table 2) (https://nextstrain.org/ncov/global) [4, 5]. The more critical RdRp amino acids V557 and D618 that directly affect the affinity of RdRp-NTP binding and stringency of base pairing are also well-preserved [35, 40, 71]. The lack of mutations in the NTP-interacting sites in RdRp suggests the plausible inhibition of RdRp by GS-441524 in VOCs (Alpha, Beta, Gamma, Delta, and Omicron variants) [74].

**Table 2:** VOC lineages with mutated GS-441524 interacting residues

NSP	Mutation	VOC code	Lineage	Characteristic mutation?*	Worldwide identified sequences	Worldwide accumulated prevalence^†^	First identified	Latest update
RdRp	None	–	–	–	–	–	–	–
NSP3	G46E	Delta	AY.25	N	132,769	1%	21 July 2020	26 February 2022
	V49I	Delta	AY.121	N	37,231	< 0.5%	24 January 2021	3 March 2022
	V49I	Delta	AY.20	N	35,647	< 0.5%	14 January 2021	7 February 2022
	I131V	Delta	B.1.617.2	N	160,901	2%	27 March 2020	7 March 2022
	A154N	Omicron	BA.1.1	N	900,337	10%	29 October 2020	21 March 2022
	A154T	Omicron	BA.1	N	1,045,934	12%	23 October 2020	17 March 2022
NSP5	None	–	–	–	–	–	–	–
NSP14	H95Y	Omicron	BA.1	N	1,045,934	12%	23 October 2020	17 March 2022

^*^Appears in more than 75% of identified sequences of the same lineage

^†^Prevalence is calculated according to 9,236,360 accumulated sequences from GISAID (Last update: GMT March 23rd, 2022 [78, 88])

All the mutations can be referred to source information in Supplementary Table S2

### Complementary Binding to NSP3 for Inactivation

NSP3 is one of the two major proteases in SARS-CoV-2 that facilitates the cleavage of the polyprotein into NSP1, 2, and 3 (Table 1) [4, 5]. The NSP3 macrodomain is conserved across coronaviruses and generally binds adenosine-5-diphosphoribose (ADP-ribose) [75, 76]. The similar sizes of ADP-ribose (substrate of NSP3) and NTP suggest that NTP may have the potential to interact with NSP3 [75, 76]. Indeed, there is in silico evidence demonstrating the interaction of GS-441524 with the macrodomain of NSP3 through hydrogen bonding and hydrophobic interaction [76, 77]. The binding mode of GS-441524 to the macrodomain is also highly similar to that of the ADP-ribose adenosine moiety [75, 76]. Moreover, GS-441524 is more structurally complementary to the ADP-ribose binding pocket in NSP3 than the adenosine substrate [51]. This suggests a secondary mechanism of inhibition by GS-441524 in addition to bioconversion to the NTP analogue.

Mutations in NSP3 may influence the interactions between GS-441524 and NSP3. As of March 20th, 2022, two different mutations (lineage significance cut-off at 10% global prevalence) in two samples of Omicron variant lineages (A154T: BA.1 and A154N: BA.1.1) were identified in an single amino acid residue of NSP3 that interacts with NTP (Table 2) (https://nextstrain.org/ncov/global) [4, 5]. The A154 residue is not one of the major amino acids highlighted in the in silico studies, and neither A154N nor A154T is a characteristic mutation of the BA.1 or BA.1.1 lineage [75, 76]. We also found several other mutations in GS-441524-NSP3 interacting sites of Delta and Omicron variant cases on Nextstrain; however, all these mutations occurred in patchy cases and with low prevalence (< 0.5–2%) (Table 2 and Supplementary Table S2). Although all the mutated residues exert relatively weak Van der Waals forces to enhance GS-441524-NSP3 interaction, two of them are in sub-lineages with over 10% global prevalence [76, 78]. Moreover, the overall number of samples with mutations in GS-441524-NSP3 interacting residues is significantly higher than in NSP5, RdRp, and NSP14. Therefore, the interactions between GS-441524 and NSP5 of VOCs would likely be affected.

### Inhibition of NSP5

NSP5 mediates the processing of NSPs at 11 cleavage sites (NSP4-11, NSP12-15) (Table 1) [24, 79]. In conjunction with NSP3, the two proteases cleave SARS-CoV-2 encode precursor polyproteins pp1a and pp1b into 16 NSPs to assemble the viral replicase complex [24]. Among crucial NSP5 residues, C145 and H164 exhibit strong hydrogen bonding with Remdesivir [80, 81]. Current data suggest H164 as an essential active site for NSP5 function, which when disrupted, may potentially halt its proteolytic activity [80]. As the structure of the interaction site in Remdesivir is conserved in GS-441524, such interactions between GS-441524 and the NSP5 active site are highly probable [81]. Thus, it is important to investigate whether mutations in the active site are observed that could potentially disrupt GS-441524-NSP5 interactions.

Similar to RdRp, since no mutation of NTP-interacting residues in NSP5 had been found as of March 20th, 2022, the interactions between GS-441524 and NSP5 of VOCs (Alpha, Beta, Gamma, Delta, and Omicron variants) would appear similar to that between GS-441524 and Wuhan wild-type NSP5 (Table 2) (https://nextstrain.org/ncov/global) [4, 5].

### Blockage of the Active Site in NSP14

As a 3′-to-5′ exoribonuclease and a guanine-N7-methyltransferase, NSP14 is a crucial component securing the replication of SARS-CoV-2 (Table 1) [82, 83]. The exoribonuclease domain of NSP14 is critical for viral replication given that mutant exoribonuclease knockout SARS-CoV-2 results in interruption of viral replication [84]. NSP10, the replicative cofactor of NSP14, stabilizes and stimulates enzymatic activities through interaction with exoribonuclease [85]. Furthermore, NSP10 has been proposed to interact with NSP12 to undergo RNA repair processes that may arise during RNA synthesis, indicating the possibility of interactions between NSP10, NSP12, and NSP14 [83]. Studies provided evidence suggesting that NSP14 interacts with NTP, where the cyano group at the 1′-ribose position of NTP fit complementarily with the active site of NSP14 exoribonuclease [40, 83]. The distorted base of NTP is predicted to prevent the proper distances for efficient two-metal ion catalysis, thus disrupting the function of exoribonuclease [40]. Due to the importance of complementarity in ensuring the effect of NTP on NSP14 activities, it is worth investigating the NSP14 mutations in VOCs to assess their influences on the potency of NTP.

As of March 20th, 2022, one mutated NSP14 residue that interacts with GS-441524 is observed on Nextstrain in a sample of the Omicron variant sub-lineage BA.1 (https://nextstrain.org/ncov/global) [4, 5]. The H95Y mutation is not characteristic for the BA.1 lineage, implying that it would not be present in all samples of the sub-lineage (Table 2). However, it is attention-worthy due to the high global prevalence (12%) of the BA.1 sub-lineage (Supplementary Table S2). Therefore, a more conservative conclusion is that the interactions of GS-441524 with NSP14 would be changed in the Omicron variant but would remain relatively conserved across the other VOCs.

## Preclinical and Clinical Studies Using GS-441524 in SARS-CoV-2 Infection

GS-441524 has shown potency in lowering SARS-CoV-2 replication in in vitro human lung Calu-3 cell infection [34], in mice with an increased viral clearance 2 days post-infection and reduced weight loss [43], and has demonstrated exceptional safety, tolerability, and pharmacokinetics in one human (case report; Cmax: 12·01 μM, surpassing the concentration required to eradicate SARS-CoV-2 in vitro [51, 52]) and several preclinical species [50]. A human study of orally administered GS-441524 for COVID-19 is underway [51, 52]. Taken together, clinical studies of GS-441524 on VOCs are of great interest, given its antiviral potentials.

## Limitations

The data for the NSP amino acid residues that interact with GS-441524 are based largely on in silico studies, and continual vigorous analysis are needed for further verification [40, 71, 77, 86]. The effect of mutations on the binding affinity of NSPs to GS-441524 remains to be monitored in emerging VOCs. The potential discrepancy may exist between the microscopic effect of the mutated amino acids and their macroscopic influence on NSP structure and protein–protein interactions. Nevertheless, our analysis provides a foundation for future clinical trial testing of GS-441524 in breakthrough VOCs. The promising results of combination therapies in recent literature also suggest that combining virus-directed and host-directed drugs may partially help to counteract the possible reduction in potency of antiviral drugs against the emerging SARS-CoV-2 variants.

## Conclusion

**Fig. 3:**
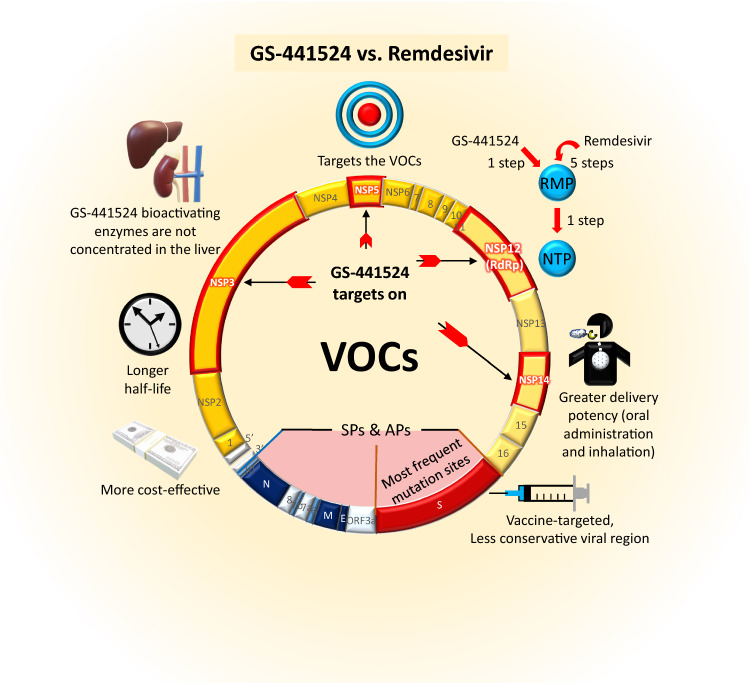
Graphical Summary of the Advantages of GS-441524 over Remdesivir against VOCs. SPs (E, M, N, S) and APs (ORF3a-10), especially S protein (Spike), are more active in forming mutations than NSPs. All COVID-19 vaccines target the most mutated parts of S protein and eventually lead to breakthrough infections in vaccinated individuals. GS-441524 and Remdesivir are both validified to target RdRp, yet GS-441524 can potentially interact with NSP3, 5, and 14, which are less mutated among VOCs. Compared to Remdesivir, GS-441524 takes fewer steps to be converted into the active metabolite NTP. GS-441524, as a precursor in the chemical synthesis of Remdesivir and with a longer half-life, is considerably more economical with less production procedures involved. NSPs, non-structural proteins; SPs, structural proteins; Aps, accessory proteins; VOCs, variants of concern

Given the recent rise of breakthrough SARS-CoV-2 cases and the emerging Alpha, Beta, Gamma, Delta, and Omicron variants that have shown spike protein mutations, there is an urgent need to examine antiviral candidates that could contain these VOCs from escaping vaccines. The major amino acid sites of NSPs (NSP3, 5, 12, and 14) that interact with the parental nucleotide GS-441524 are not altered in the emerging VOCs. As such, we believe that the ready-to-use GS-441524 is a potential antiviral approach against the breakthrough VOCs (Fig. 3).

## Supplementary Information

Below is the link to the electronic supplementary material.

Supplementary file 1 (DOCX 15 kb)

## Data Availability

Not applicable.

## Abbreviations

ACE2: Angiotensin-converting enzyme 2
APs: Accessory proteins
CES1: Carboxylesterase 1
COVID-19: Coronavirus disease 2019
CTSA: Cathepsin A
FIASMA: Functional Inhibitors of Acid Sphingomyelinase
HINT1: Histidine triad nucleotide-binding protein 1
NSPs: Non-structural proteins
NTP: Nucleoside triphosphate
RBD: Receptor-binding domain
RdRp: RNA-dependent RNA polymerase
SARS-CoV-2: Severe acute respiratory syndrome coronavirus 2
SPs: Structural proteins
vdW: Van der Waals
VOCs: Variants of concern

## References

[1] Auwaerter P. Coronavirus COVID-19 (SARS-CoV-2). The Johns Hopkins University; 2021. Available from: https://www.hopkinsguides.com/hopkins/view/Johns_Hopkins_ABX_Guide/540747/all/Coronavirus_COVID_19__SARS_CoV_2_

[2] Dong E, Du H, Gardner L (2020). An interactive web-based dashboard to track COVID-19 in real time. Lancet Infect Dis Lancet Publ Group.

[3] Coronavirus COVID-19 (2019-nCoV). Available from: https://gisanddata.maps.arcgis.com/apps/dashboards/bda7594740fd40299423467b48e9ecf6

[4] Nextstrain/ncov/global. Available from: https://nextstrain.org/ncov/global

[5] Hadfield J, Megill C, Bell SM, Huddleston J, Potter B, Callender C (2018). NextStrain: real-time tracking of pathogen evolution. Bioinformatics.

[6] Stanford Coronavirus Antiviral & Resistance Database. SARS-CoV-2 Variants. Available from: https://covdb.stanford.edu/page/mutation-viewer/#sec_delta

[7] Latif AA, Mullen JL, Alkuzweny M, Tsueng G, Cano M, Haag E, et al. B.1.1.7 Lineage Report. 2021. Available from: https://outbreak.info/situation-reports?pango=B.1.1.7

[8] Latif AA, Mullen JL, Alkuzweny M, Tsueng G, Cano M, Haag E, et al. B.1.351 Lineage Report. Available from: https://outbreak.info/situation-reports?pango=B.1.351

[9] Latif AA, Mullen JL, Alkuzweny M, Tsueng G, Cano M, Haag E, et al. P.1 Lineage Report. Available from: https://outbreak.info/situation-reports?pango=P.1

[10] Latif AA, Mullen JL, Alkuzweny M, Tsueng G, Cano M, Haag E, et al. B.1.617.2 Lineage Report. 2021. Available from: https://outbreak.info/situation-reports?pango=B.1.617.2

[11] Latif AA, Mullen JL, Alkuzweny M, Tsueng G, Cano M, Haag E, et al. B.1.1.529 Lineage Report. 2021.

[12] Garcia-Beltran WF, Lam EC, St. Denis K, Nitido AD, Garcia ZH, Hauser BM, et al. Multiple SARS-CoV-2 variants escape neutralization by vaccine-induced humoral immunity. Cell. Elsevier B.V.; 2021;184:2372–2383.e9. Available from: https://pubmed.ncbi.nlm.nih.gov/33743213/10.1016/j.cell.2021.03.013 PMC7953441 33743213

[13] Fisman DN, Tuite AR. Evaluation of the relative virulence of novel SARS-CoV-2 variants: a retrospective cohort study in Ontario, Canada. CMAJ. CMAJ; 2021;193:E1619–25. Available from: https://www.cmaj.ca/content/193/42/E1619 10.1503/cmaj.211248 PMC8562985 34610919

[14] Pulliam JRC, Schalkwyk C van, Govender N, Gottberg A von, Cohen C, Groome MJ, et al. Increased risk of SARS-CoV-2 reinfection associated with emergence of the Omicron variant in South Africa. medRxiv. Cold Spring Harbor Laboratory Press; 2021;2021.11.11.21266068. Available from: 10.1101/2021.11.11.21266068v2

[15] Chen J, Wei G-W. Omicron BA.2 (B.1.1.529.2): high potential to becoming the next dominating variant. ArXiv. ArXiv; 2022; Available from: https://pubmed.ncbi.nlm.nih.gov/35169598/

[16] Wang Y, Zhang L, Li Q, Liang Z, Li T, Liu S, et al. The significant immune escape of pseudotyped SARS-CoV-2 variant Omicron. Emerg Microbes Infect. Emerg Microbes Infect; 2022;11:1–5. Available from: https://pubmed.ncbi.nlm.nih.gov/34890524/10.1080/22221751.2021.2017757 PMC8725892 34890524

[17] Nyberg T, Ferguson NM, Nash SG, Webster HH, Flaxman S, Andrews N, et al. Comparative analysis of the risks of hospitalisation and death associated with SARS-CoV-2 omicron (B.1.1.529) and delta (B.1.617.2) variants in England: a cohort study. Lancet. Elsevier; 2022;0. Available from: http://www.thelancet.com/article/S0140673622004627/fulltext 10.1016/S0140-6736(22)00462-7 PMC8926413 35305296

[18] Garcia-Beltran WF, St. Denis KJ, Hoelzemer A, Lam EC, Nitido AD, Sheehan ML, et al. mRNA-based COVID-19 vaccine boosters induce neutralizing immunity against SARS-CoV-2 Omicron variant. Cell. Cell; 2022;185:457–466.e4. Available from: https://pubmed.ncbi.nlm.nih.gov/34995482/10.1016/j.cell.2021.12.033 PMC8733787 34995482

[19] Antonelli M, Penfold RS, Merino J, Sudre CH, Molteni E, Berry S, et al. Risk factors and disease profile of post-vaccination SARS-CoV-2 infection in UK users of the COVID Symptom Study app: a prospective, community-based, nested, case-control study. Lancet Infect Dis. Elsevier; 2021. 10.1016/S1473-3099(21)00460-6 PMC8409907 34480857

[20] Brown C, Vostok J, Johnson H, Burns M, Gharpure R, Sami S, et al. Outbreak of SARS-CoV-2 Infections, Including COVID-19 Vaccine Breakthrough Infections, Associated with Large Public Gatherings—Barnstable County, Massachusetts, July 2021. MMWR Morb Mortal Wkly Rep. MMWR Morb Mortal Wkly Rep; 2021;70:1059–62. Available from: https://pubmed.ncbi.nlm.nih.gov/34351882/10.15585/mmwr.mm7031e2 PMC8367314 34351882

[21] Chia PY, Xiang Ong SW, Chiew CJ, Ang LW, Chavatte J-M, Mak T-M, et al. Virological and serological kinetics of SARS-CoV-2 Delta variant vaccine-breakthrough infections: a multi-center cohort study. Clin Microbiol Infect. Clin Microbiol Infect; 2021; Available from: https://pubmed.ncbi.nlm.nih.gov/34826623/10.1016/j.cmi.2021.11.010 PMC8608661 34826623

[22] Ahmad L. Implication of SARS-CoV-2 Immune Escape Spike Variants on Secondary and Vaccine Breakthrough Infections. Front Immunol. Frontiers; 2021;0:4563. Available from: 10.3389/fimmu.2021.742167/full 10.3389/fimmu.2021.742167 PMC8596465 34804022

[23] Callaway E (2021). Delta coronavirus variant: scientists brace for impact. Nature NLM (Medline).

[24] Romano M, Ruggiero A, Squeglia F, Maga G, Berisio R. A Structural View of SARS-CoV-2 RNA Replication Machinery: RNA Synthesis, Proofreading and Final Capping. Cells. NLM (Medline); 2020;9:1267. Available from: https://www.pmc/articles/PMC7291026/10.3390/cells9051267 PMC7291026 32443810

[25] Khateeb J, Li Y, Zhang H. Emerging SARS-CoV-2 variants of concern and potential intervention approaches. Crit Care. Crit Care; 2021;25. Available from: https://pubmed.ncbi.nlm.nih.gov/34253247/10.1186/s13054-021-03662-x PMC8274962 34253247

[26] Islam MR, Hoque MN, Rahman MS, Alam ASMRU, Akther M, Puspo JA (2020). Genome-wide analysis of SARS-CoV-2 virus strains circulating worldwide implicates heterogeneity. Sci Rep Nat Res.

[27] Government of Canada. COVID-19 Drugs and vaccines—Canada.ca. 2021. Available from: https://www.canada.ca/en/health-canada/services/drugs-health-products/covid19-industry/drugs-vaccines-treatments.html

[28] Gómez CE, Perdiguero B, Esteban M. Emerging SARS-COV-2 variants and impact in global vaccination programs against SARS-COV-2/COVID-19. Vaccines. MDPI AG; 2021;9:1–13. Available from: https://www.pmc/articles/PMC7999234/10.3390/vaccines9030243 PMC7999234 33799505

[29] The effects of virus variants on COVID-19 vaccines. Available from: https://www.who.int/news-room/feature-stories/detail/the-effects-of-virus-variants-on-covid-19-vaccines

[30] Antiviral Therapy | COVID-19 Treatment Guidelines. Available from: https://www.covid19treatmentguidelines.nih.gov/antiviral-therapy/

[31] Iacob S, Iacob DG. SARS-CoV-2 Treatment Approaches: Numerous Options, No Certainty for a Versatile Virus. Front Pharmacol. Frontiers Media S.A.; 2020;11:1224. Available from: https://pubmed.ncbi.nlm.nih.gov/32982720/10.3389/fphar.2020.01224 PMC7479232 32982720

[32] Pardi N, Weissman D. Development of vaccines and antivirals for combating viral pandemics. Nat Biomed Eng Nat Res. 2020;4:1128–33. Available from: www.nature.com/natbiomedeng 10.1038/s41551-020-00658-w PMC8336060 33293724

[33] Yan VC, Muller FL. Advantages of the Parent Nucleoside GS-441524 over Remdesivir for Covid-19 Treatment. ACS Med Chem Lett. American Chemical Society; 2020;11:1361–6. Available from: https://www.pmc/articles/PMC7315846/10.1021/acsmedchemlett.0c00316 PMC7315846 32665809

[34] Pruijssers AJ, George AS, Schäfer A, Leist SR, Gralinksi LE, Dinnon KH, et al. Remdesivir Inhibits SARS-CoV-2 in Human Lung Cells and Chimeric SARS-CoV Expressing the SARS-CoV-2 RNA Polymerase in Mice. Cell Rep. Elsevier B.V.; 2020;32:107940. Available from: 10.1016/j.celrep.2020.107940 10.1016/j.celrep.2020.107940 PMC7340027 32668216

[35] RCSB PDB-7L1F: SARS-CoV-2 RdRp in complex with 4 Remdesivir monophosphate. Available from: https://www.rcsb.org/structure/7L1F

[36] Beigel JH, Tomashek KM, Dodd LE, Mehta AK, Zingman BS, Kalil AC, et al. Remdesivir for the Treatment of Covid-19—Final Report. N Engl J Med. Massachusetts Medical Society; 2020;383:1813–26. Available from: 10.1056/nejmoa2007764 10.1056/NEJMoa2007764 PMC7262788 32445440

[37] Repurposed Antiviral Drugs for Covid-19—Interim WHO Solidarity Trial Results. N Engl J Med. Massachusetts Medical Society; 2021;384:497–511. Available from: https://pubmed.ncbi.nlm.nih.gov/33264556/10.1056/NEJMoa2023184 PMC7727327 33264556

[38] Ohl ME, Miller DR, Lund BC, Kobayashi T, Richardson Miell K, Beck BF, et al. Association of Remdesivir Treatment With Survival and Length of Hospital Stay Among US Veterans Hospitalized With COVID-19. JAMA Netw Open. American Medical Association; 2021;4:e2114741–e2114741. Available from: https://jamanetwork.com/journals/jamanetworkopen/fullarticle/2781959 10.1001/jamanetworkopen.2021.14741 PMC8283561 34264329

[39] Wang Y, Zhang D, Du G, Du R, Zhao J, Jin Y, et al. Remdesivir in adults with severe COVID-19: a randomised, double-blind, placebo-controlled, multicentre trial. Lancet. Lancet Publishing Group; 2020;395:1569–78. Available from: http://www.thelancet.com/article/S0140673620310229/fulltext 10.1016/S0140-6736(20)31022-9 PMC7190303 32423584

[40] Shannon A, Le NTT, Selisko B, Eydoux C, Alvarez K, Guillemot JC, et al. Remdesivir and SARS-CoV-2: Structural requirements at both nsp12 RdRp and nsp14 Exonuclease active-sites. Antiviral Res. Elsevier B.V.; 2020;178:104793. Available from: https://pubmed.ncbi.nlm.nih.gov/32283108/10.1016/j.antiviral.2020.104793 PMC7151495 32283108

[41] Hacisuleyman E, Hale C, Saito Y, Blachere NE, Bergh M, Conlon EG, et al. Vaccine Breakthrough Infections with SARS-CoV-2 Variants. Massachusetts Medical Society; 2021;384:2212–8. Available from: 10.1056/NEJMoa2105000 10.1056/NEJMoa2105000 PMC8117968 33882219

[42] Farinholt T, Doddapaneni H, Qin X, Menon V, Meng Q, Metcalf G, et al. Transmission event of SARS-CoV-2 delta variant reveals multiple vaccine breakthrough infections. BMC Med. BioMed Central Ltd; 2021;19:1–6. Available from: 10.1186/s12916-021-02103-4 10.1186/s12916-021-02103-4 PMC8483940 34593004

[43] Li Y, Cao L, Li G, Cong F, Li Y, Sun J, et al. Remdesivir Metabolite GS-441524 Effectively Inhibits SARS-CoV-2 Infection in Mouse Models. J Med Chem. American Chemical Society; 2021; Available from: 10.1021/acs.jmedchem.0c01929 10.1021/acs.jmedchem.0c01929 33523654

[44] Sciences Inc G. Fact sheet for healthcare providers emergency use authorization (EUA) of Veklury^®^ (Remdesivir) for hospitalized pediatric patients weighing 3.5 kg to less than 40 kg or hospitalized pediatric patients less than 12 years of age weighing at least 3.5 kg. Available from: https://www.fda.gov/emergency-preparedness-and-response/mcm-legal-

[45] Schafer A, Martinez DR, Won JJ, Meganck RM, Moreira FR, Brown AJ, et al. Therapeutic treatment with an oral prodrug of the remdesivir parental nucleoside is protective against SARS-CoV-2 pathogenesis in mice. Sci Transl Med. American Association for the Advancement of Science; 2022;14:3410. Available from: 10.1126/scitranslmed.abm3410 10.1126/scitranslmed.abm3410 PMC8995034 35315683

[46] Di L. The Impact of Carboxylesterases in Drug Metabolism and Pharmacokinetics. Curr Drug Metab. Bentham Science Publishers; 2018;19:91. 10.2174/1389200219666180821094502 PMC6635651 30129408

[47] Wang D, Zou L, Jin Q, Hou J, Ge G, Yang L (2018). Human carboxylesterases: a comprehensive review. Acta Pharm Sin B Elsevier.

[48] Cox RM, Wolf JD, Lieber CM, Sourimant J, Lin MJ, Babusis D, et al. Oral prodrug of remdesivir parent GS-441524 is efficacious against SARS-CoV-2 in ferrets. Nat Commun. Springer US; 2021;12:6415. 10.1038/s41467-021-26760-4 PMC8571282 34741049

[49] Xu Y, Barauskas O, Kim C, Babusis D, Murakami E, Kornyeyev D, et al. Off-Target In Vitro Profiling Demonstrates that Remdesivir Is a Highly Selective Antiviral Agent. Antimicrob Agents Chemother. Antimicrob Agents Chemother; 2021;65. Available from: https://pubmed.ncbi.nlm.nih.gov/33229429/10.1128/AAC.02237-20 PMC7849018 33229429

[50] GS-441524 Pharmacokinetic (PK) Studies. Available from: https://opendata.ncats.nih.gov/covid19/GS-441524

[51] First-in-Human Study of Orally Administered GS-441524 for COVID-19—Tabular View—ClinicalTrials.gov. Available from: https://clinicaltrials.gov/ct2/show/record/NCT04859244

[52] Yan V. First-in-Woman Safety, Tolerability, and Pharmacokinetics of Orally Administered GS-441524: A Broad-Spectrum Antiviral Treatment for COVID-19. OSF Preprints; Available from: https://osf.io/am5s8/

[53] Hill A, Wang J, Levi J, Heath K, Fortunak J (2020). Minimum costs to manufacture new treatments for COVID-19. J Virus Erad.

[54] Reis G, Moreira-Silva EA dos S, Silva DCM, Thabane L, Milagres AC, Ferreira TS, et al. Effect of early treatment with fluvoxamine on risk of emergency care and hospitalisation among patients with COVID-19: the TOGETHER randomised, platform clinical trial. Lancet Glob Heal. Elsevier; 2021;0. Available from: http://www.thelancet.com/article/S2214109X21004484/fulltext 10.1016/S2214-109X(21)00448-4 PMC8550952 34717820

[55] Lenze EJ, Mattar C, Zorumski CF, Stevens A, Schweiger J, Nicol GE, et al. Fluvoxamine vs. Placebo and Clinical Deterioration in Outpatients With Symptomatic COVID-19: A Randomized Clinical Trial. JAMA. American Medical Association; 2020;324:2292–300. Available from: https://jamanetwork.com/journals/jama/fullarticle/2773108 10.1001/jama.2020.22760 PMC7662481 33180097

[56] Schloer S, Brunotte L, Mecate-Zambrano A, Zheng S, Tang J, Ludwig S, et al. Drug synergy of combinatory treatment with remdesivir and the repurposed drugs fluoxetine and itraconazole effectively impairs SARS‐CoV‐2 infection in vitro. Br J Pharmacol [Internet]. Wiley-Blackwell; 2021 [cited 2022 October 13];178:2339. Available from: https://www.pmc/articles/PMC8251190/10.1111/bph.15418 PMC8251190 33825201

[57] Brunotte L, Zheng S, Mecate-Zambrano A, Tang J, Ludwig S, Rescher U, et al. Combination therapy with fluoxetine and the nucleoside analog gs-441524 exerts synergistic antiviral effects against different SARS-COV-2 variants in vitro. Pharmaceutics [Internet]. MDPI; 2021 [cited 2022 October 13];13. Available from: https://www.pmc/articles/PMC8466181/10.3390/pharmaceutics13091400 PMC8466181 34575474

[58] Hoertel N, Sánchez-Rico M, Gulbins E, Kornhuber J, Carpinteiro A, Abellán M, et al. Association between FIASMA psychotropic medications and reduced risk of intubation or death in individuals with psychiatric disorders hospitalized for severe COVID-19: an observational multicenter study. Transl Psychiatry 2022 121 [Internet]. Nature Publishing Group; 2022 [cited 2022 October 16];12:1–11. Available from: https://www.nature.com/articles/s41398-022-01804-5 10.1038/s41398-022-01804-5 PMC8892828 35241663

[59] Schloer S, Brunotte L, Goretzko J, Mecate-Zambrano A, Korthals N, Gerke V, et al. Targeting the endolysosomal host-SARS-CoV-2 interface by clinically licensed functional inhibitors of acid sphingomyelinase (FIASMA) including the antidepressant fluoxetine. Emerg Microbes Infect [Internet]. Emerg Microbes Infect; 2020 [cited 2022 October 13];9:2245–55. Available from: https://pubmed.ncbi.nlm.nih.gov/32975484/10.1080/22221751.2020.1829082 PMC7594754 32975484

[60] Fred SM, Kuivanen S, Ugurlu H, Casarotto PC, Levanov L, Saksela K, et al. Antidepressant and Antipsychotic Drugs Reduce Viral Infection by SARS-CoV-2 and Fluoxetine Shows Antiviral Activity Against the Novel Variants in vitro. Front Pharmacol [Internet]. Front Pharmacol; 2022 [cited 2022 October 13];12. Available from: https://pubmed.ncbi.nlm.nih.gov/35126106/10.3389/fphar.2021.755600 PMC8809408 35126106

[61] Seftel D, Boulware DR. Prospective Cohort of Fluvoxamine for Early Treatment of Coronavirus Disease 19. Open Forum Infect Dis [Internet]. Oxford Academic; 2021 [cited 2022 October 16];8. Available from: https://academic.oup.com/ofid/article/8/2/ofab050/6124100 10.1093/ofid/ofab050 PMC7888564 33623808

[62] Fritz BA, Hoertel N, Lenze EJ, Jalali F, Reiersen AM. Association between antidepressant use and ED or hospital visits in outpatients with SARS-CoV-2. Transl Psychiatry [Internet]. Transl Psychiatry; 2022 [cited 2022 October 13];12. Available from: https://pubmed.ncbi.nlm.nih.gov/35995770/10.1038/s41398-022-02109-3 PMC9395392 35995770

[63] Zhao H, Lu L, Peng Z, Chen LL, Meng X, Zhang C, et al. SARS-CoV-2 Omicron variant shows less efficient replication and fusion activity when compared with Delta variant in TMPRSS2-expressed cells [Internet]. Taylor & Francis; 2022 [cited 2022 October 16];11:277–83. Available from: 10.1080/22221751.2021.2023329 10.1080/22221751.2021.2023329 PMC8774049 34951565

[64] Kalil AC, Patterson TF, Mehta AK, Tomashek KM, Wolfe CR, Ghazaryan V, et al. Baricitinib plus Remdesivir for Hospitalized Adults with Covid-19. N Engl J Med [Internet]. N Engl J Med; 2021 [cited 2022 October 14];384:795–807. Available from: https://pubmed.ncbi.nlm.nih.gov/33306283/10.1056/NEJMoa2031994 PMC7745180 33306283

[65] Schreiber A, Ambrosy B, Planz O, Schloer S, Rescher U, Ludwig S. The MEK1/2 Inhibitor ATR-002 (Zapnometinib) Synergistically Potentiates the Antiviral Effect of Direct-Acting Anti-SARS-CoV-2 Drugs. Pharmaceutics [Internet]. Pharmaceutics; 2022 [cited 2022 October 14];14. Available from: https://pubmed.ncbi.nlm.nih.gov/36145524/10.3390/pharmaceutics14091776 PMC9506552 36145524

[66] Le Corre P, Loas G. Difficulty in Repurposing Selective Serotonin Reuptake Inhibitors and Other Antidepressants with Functional Inhibition of Acid Sphingomyelinase in COVID-19 Infection. Front Pharmacol. Frontiers Media S.A.; 2022;13:350. 10.3389/fphar.2022.849095 PMC8927035 35308205

[67] Carpinteiro A, Gripp B, Hoffmann M, Pöhlmann S, Hoertel N, Edwards MJ, et al. Inhibition of acid sphingomyelinase by ambroxol prevents SARS-CoV-2 entry into epithelial cells. J Biol Chem [Internet]. American Society for Biochemistry and Molecular Biology Inc.; 2021 [cited 2022 October 25];296:100701. Available from: http://www.jbc.org/article/S0021925821004907/fulltext 10.1016/j.jbc.2021.100701 PMC8062550 33895135

[68] Kirchdoerfer RN, Ward AB. Structure of the SARS-CoV nsp12 polymerase bound to nsp7 and nsp8 co-factors. Nat Commun. Nature Publishing Group; 2019;10:1–9. Available from: 10.1038/s41467-019-10280-3 10.1038/s41467-019-10280-3 PMC6538669 31138817

[69] Yin W, Mao C, Luan X, Shen DD, Shen Q, Su H, et al. Structural basis for inhibition of the RNA-dependent RNA polymerase from SARS-CoV-2 by remdesivir. Science (80–). American Association for the Advancement of Science; 2020;368:1499–504. Available from: http://science.sciencemag.org/10.1126/science.abc1560 PMC7199908 32358203

[70] Gordon CJ, Tchesnokov EP, Feng JY, Porter DP, Götte M. The antiviral compound remdesivir potently inhibits RNAdependent RNA polymerase from Middle East respiratory syndrome coronavirus. J. Biol. Chem. American Society for Biochemistry and Molecular Biology Inc.; 2020. p. 4773–9. Available from: https://pubmed.ncbi.nlm.nih.gov/32094225/10.1074/jbc.AC120.013056 PMC7152756 32094225

[71] Zhang L, Zhou R. Structural Basis of the Potential Binding Mechanism of Remdesivir to SARS-CoV-2 RNA-Dependent RNA Polymerase. J Phys Chem B. American Chemical Society; 2020;124:6955–62. Available from: 10.1021/acs.jpcb.0c04198 10.1021/acs.jpcb.0c04198 32521159

[72] Li R, Liclican A, Xu Y, Pitts J, Niu C, Zhang J, et al. Key Metabolic Enzymes Involved in Remdesivir Activation in Human Lung Cells. Antimicrob Agents Chemother. Antimicrob Agents Chemother; 2021;65. Available from: https://pubmed.ncbi.nlm.nih.gov/34125594/10.1128/AAC.00602-21 PMC8370248 34125594

[73] Agostini ML, Andres EL, Sims AC, Graham RL, Sheahan TP, Lu X, et al. Coronavirus Susceptibility to the Antiviral Remdesivir (GS-5734) Is Mediated by the Viral Polymerase and the Proofreading Exoribonuclease. MBio. mBio; 2018;9. Available from: https://pubmed.ncbi.nlm.nih.gov/29511076/10.1128/mBio.00221-18 PMC5844999 29511076

[74] Do TND, Donckers K, Vangeel L, Chatterjee AK, Gallay PA, Bobardt MD, et al. A robust SARS-CoV-2 replication model in primary human epithelial cells at the air liquid interface to assess antiviral agents. Antiviral Res. Elsevier; 2021;192:105122. 10.1016/j.antiviral.2021.105122 PMC8233549 34186107

[75] Jung LS, Gund TM, Narayan M. Comparison of Binding Site of Remdesivir and Its Metabolites with NSP12-NSP7-NSP8, and NSP3 of SARS CoV-2 Virus and Alternative Potential Drugs for COVID-19 Treatment. Protein J. Springer; 2020;39:619–30. Available from: https://pubmed.ncbi.nlm.nih.gov/33185784/10.1007/s10930-020-09942-9 PMC7662030 33185784

[76] Ni X, Schröder M, Olieric V, Sharpe ME, Hernandez-Olmos V, Proschak E, et al. Structural Insights into Plasticity and Discovery of Remdesivir Metabolite GS-441524 Binding in SARS-CoV-2 Macrodomain. ACS Med Chem Lett. American Chemical Society; 2021;12:603–9. Available from: 10.1021/acsmedchemlett.0c00684 10.1021/acsmedchemlett.0c00684 PMC7986975 33850605

[77] RCSB PDB-7BF6: Crystal structure of SARS-CoV-2 macrodomain in complex with remdesivir metabolite GS-441524. Available from: https://www.rcsb.org/structure/7BF6

[78] Outbreak.info. Available from: https://outbreak.info/?

[79] Stobart CC, Sexton NR, Munjal H, Lu X, Molland KL, Tomar S, et al. Chimeric Exchange of Coronavirus nsp5 Proteases (3CLpro) Identifies Common and Divergent Regulatory Determinants of Protease Activity. J Virol. American Society for Microbiology; 2013;87:12611–8. Available from: https://jvi.asm.org/content/early/2013/09/05/JVI.02050-13 10.1128/JVI.02050-13 PMC3838113 24027335

[80] Xu C, Ke Z, Liu C, Wang Z, Liu D, Zhang L, et al. Systemic in Silico Screening in Drug Discovery for Coronavirus Disease (COVID-19) with an Online Interactive Web Server. J Chem Inf Model. American Chemical Society; 2020;60:5735–45. Available from: https://www.pmc/articles/PMC7460831/10.1021/acs.jcim.0c00821 32786695

[81] Naik VR, Munikumar M, Ramakrishna U, Srujana M, Goudar G, Naresh P, et al. Remdesivir (GS-5734) as a therapeutic option of 2019-nCOV main protease–in silico approach. J Biomol Struct Dyn. Taylor and Francis Ltd.; 2020;1–14. Available from: https://www.tandfonline.com/action/journalInformation?journalCode=tbsd20 10.1080/07391102.2020.1781694 PMC7332877 32568620

[82] Khalaf K, Papp N, Chou JTT, Hana D, Mackiewicz A, Kaczmarek M. SARS-CoV-2: Pathogenesis, and Advancements in Diagnostics and Treatment. Front Immunol. Frontiers Media S.A.; 2020;11:2462. 10.3389/fimmu.2020.570927 PMC7573101 33123144

[83] Ogando NS, Zevenhoven-Dobbe JC, van der Meer Y, Bredenbeek PJ, Posthuma CC, Snijder EJ. The Enzymatic Activity of the nsp14 Exoribonuclease Is Critical for Replication of MERS-CoV and SARS-CoV-2. J Virol. American Society for Microbiology; 2020;94:e01246–20. Available from: 10.1128/JVI 10.1128/JVI.01246-20 PMC7654266 32938769

[84] Min JS, Kim G-W, Kwon S, Jin Y-H. A Cell-Based Reporter Assay for Screening Inhibitors of MERS Coronavirus RNA-Dependent RNA Polymerase Activity. J Clin Med. MDPI AG; 2020;9:2399. Available from: https://pubmed.ncbi.nlm.nih.gov/32727069/10.3390/jcm9082399 PMC7465106 32727069

[85] Ma Y, Wu L, Shaw N, Gao Y, Wang J, Sun Y, et al. Structural basis and functional analysis of the SARS coronavirus nsp14-nsp10 complex. Proc Natl Acad Sci U S A. National Academy of Sciences; 2015;112:9436–41. Available from: 10.1073/pnas.1508686112 10.1073/pnas.1508686112 PMC4522806 26159422

[86] Kannan SR, Spratt AN, Quinn TP, Heng X, Lorson CL, Sönnerborg A, et al. Infectivity of SARS-CoV-2: there Is Something More than D614G? J Neuroimmune Pharmacol. Springer; 2020;15:574–7. Available from: https://pubmed.ncbi.nlm.nih.gov/32930936/10.1007/s11481-020-09954-3 PMC7490321 32930936

[87] Schuller M, Correy GJ, Gahbauer S, Fearon D, Wu T, Díaz RE, et al. Fragment binding to the Nsp3 macrodomain of SARS-COV-2 identified through crystallographic screening and computational docking. Sci Adv. American Association for the Advancement of Science; 2021;7. 10.1126/sciadv.abf8711 PMC8046379 33853786

[88] Elbe S, Buckland-Merrett G (2017). Data, disease and diplomacy: GISAID’s innovative contribution to global health. Glob Challenges Wiley.

