# Supplementary material for: The Potential Protective Role of GS-441524, a Metabolite of the Prodrug Remdesivir, in Vaccine Breakthrough SARS-CoV-2 Infections
Source: Intensive Care Res. 2022 Nov 9;2(3-4):49–60. doi: 10.1007/s44231-022-00021-4 (PMC9645326; doi:10.1007/s44231-022-00021-4)
Supplement: Supplementary file 1 — (DOCX 15 kb) [file 44231_2022_21_MOESM1_ESM.docx]

| **Supplementary Table 1. Summary of Non-Structural Proteins (NSP), critical Spike S1 domain and Nucleocapsid mutations in VOCs** [6–11] | | |
| --- | --- | --- |
| **SARS-CoV-2 variants** | **NSP (ORF1ab) mutations** | **Critical Spike S1 mutations and Nucleocapsid mutations** |
| **B.1.1.7** [7]  **(Alpha variant)** | NSP3: T183I, A890D, I1412T  NSP6: SGF106-108Δ  RdRp: P323L* | S: N501Y, D614G*, P681H  N: R203K, G204R |
| **B.1.351** [8]  **(Beta variant)** | NSP2: T85I  NSP3: K837N  NSP4: H26Y, S137L  NSP5: K90R  NSP6: SGF106-108Δ  RdRp: D135Y, P323L*  NSP13: T588I | S: K417N, E484K, N501Y, D614G* |
| **P.1** [9]  **(Gamma variant)** | NSP3: S370L, K977Q  NSP6: SGF106-108Δ  RdRp: P323L*  NSP13: E341D | S: K417T, E484K, N501Y, D614G*  N: R203K, G204R |
| **B.1.617.2** [10]  **(Delta variant)** | NSP3: A488S, P1228L, P1469S  NSP4: V167L, T492I  NSP6: T77A  RdRp: P323L*, G671S  NSP13: P77L  NSP14: A394V | S: L452R, T478K, E484Q, D614G*, P681R  N: R203M |
| **B.1.1.529** [11]  **(Omicron variant)** | NSP3: K38R, V1069I, S1265Δ, L1266I, A1892T  NSP4: T492I  NSP5: P132H  NSP6: LSG105-107Δ, A189V  RdRp: P323L*  NSP14: I42V | S: K417N, G446S, T478K, E484A, Q493K, G496S, Q498R, N501Y, Y505H, D614G*, H655Y, P681H  N: R203K, G204R   \|  \| \| --- \| |
| *: *Amino* *acid mutations that are present in all VOCs*  *Notes: none of the major mutations in RdRp, NSP3, NSP5, or NSP14 reported above overlap with the amino acid residues that interact with GS-441524.* | | |

| **Supplementary Table 2. Source information of VOC samples with GS-441524-NSP mutations** | | | | | | |
| --- | --- | --- | --- | --- | --- | --- |
| Mutation | Site reported on Nextstrain | GISAID EPI ISL | VOC code | PANGO Lineage | Collection date | Country/ region |
| NSP3: G46E | ORF1a: G1068E | EPI_ISL_5053122  (hCoV-19/USA/MS-UMMC-M1360A5-514652/2021) | Delta | AY.25 | 2021-09-24 | USA |
| NSP3: V49I | ORF1a: V1071I | EPI_ISL_7926645  (hCoV-19/Slovenia/23-031680-MB/2021) | Delta | AY.121 | 2021-11-26 | Slovenia |
| NSP3: V49I | ORF1a: V1071I | EPI_ISL_7853150  (hCoV-19/Mexico/GUA_LANGEBIO_IMSS_3985/2021) | Delta | AY.20 | 2021-11-21 | Mexico |
| NSP3: I131V | ORF1a: I1153V | EPI_ISL_8298670  (hCoV-19/Cameroon/CPC-21v-37297/2021) | Delta | B.1.617.2 | 2021-07-16 | Cameroon |
| NSP3: A154N | ORF1a: A1176N | EPI_ISL_9070815  (hCoV-19/Guatemala/INC-LNS-159/2022) | Omicron | BA.1 | 2022-01-01 | Guatemala |
| NSP3: A154T | ORF1a: A1176T | EPI_ISL_11289696  (hCoV-19/Peru/PIU-INS-14814/2022) | Omicron | BA.1 | 2022-01-12 | Peru |
| NSP14: H95Y | ORF1b: H1619Y | EPI_ISL_10974772  (hCoV-19/Australia/QLD0x00CA3F/2022) | Omicron | BA.1 | 2022-02-07 | Australia |
| *Amino acid residues that have mutations in VOCs reported on Nextstrain as of March 20^th^, 2022.* | | | | | | |
